# Atypical presentation of hepatic visceral larva migrans mimicking cancer and associated with ADAMTS13 deficiency–mediated thrombotic microangiopathy: A first report from Reunion Island

**DOI:** 10.1371/journal.pntd.0005617

**Published:** 2017-07-20

**Authors:** Simon Bonnefond, Aurélie Foucher, Patricia Zunic, Gautier Hoarau, Jean-François Magnaval

**Affiliations:** 1 Department of Internal Medicine and Infectious Diseases, Centre Hospitalier Universitaire de la Réunion Saint Pierre, Reunion Island, France; 2 Department of Hematology, Centre Hospitalier Universitaire de la Reunion Saint Pierre, Reunion Island, France; 3 Department of Microbiology, Centre Hospitalier Universitaire de la Reunion Saint Pierre, Reunion Island, France; 4 Department of Medical Parasitology, Faculty of Medicine Purpan, Toulouse, France; University of Queensland, AUSTRALIA

## Introduction

Human toxocariasis is a neglected soil-transmitted helminth infection from dogs and cats caused by larvae of *Toxocara canis* or *T*. *cati*, respectively. Eggs passed in hosts’ feces embryonate in the soil. Humans become infected following ingestion of embryonated eggs, and the major risk factors are poverty, contact with infected dogs and/or cats, geophagia, and consumption of contaminated raw vegetables [1,2]. Ingested eggs hatch in the duodenum, and the released larvae penetrate the mucosa, migrate to the liver via the portal circulation, follow vascular channels to the lungs, then enter the systemic circulation and finally get arrested in the tissues, thus achieving the so-called somatic cycle [1]. The spectrum of toxocaral disease encompasses various forms, including generalized visceral larva migrans (VLM) [3], covert toxocariasis, and compartmentalized (ocular and neurological toxocariasis) [4]. Among the generalized forms, covert toxocariasis, which represents the majority of the cases, is typically a self-limited, benign disease whereas VLM, which always has severe and sometimes life-threatening presentation, is quite uncommon, at least in Westernized countries. To date, the highest level of seroprevalence (93.9%) has been found in La Reunion Island among subjects over 15 years old [5].

Whether helminthiases can protect against the occurrence of autoimmune diseases or, conversely, can boost autoimmunity is still a matter of debate [6]. Should only the second consequence be considered, toxocariasis has been unequivocally found as triggering an autoimmune reaction in only 5 patients [7–11]. We report the case of a 61-year-old man living in Reunion Island who suffered from toxocariasis with significant liver involvement associated with an autoimmune thrombotic thrombocytopenic purpura (TTP) due to the presence of anti-ADAMTS13 autoantibodies.

## Ethics statement

Oral and written consent were obtained from the patient.

## Case presentation

**Fig 1:**
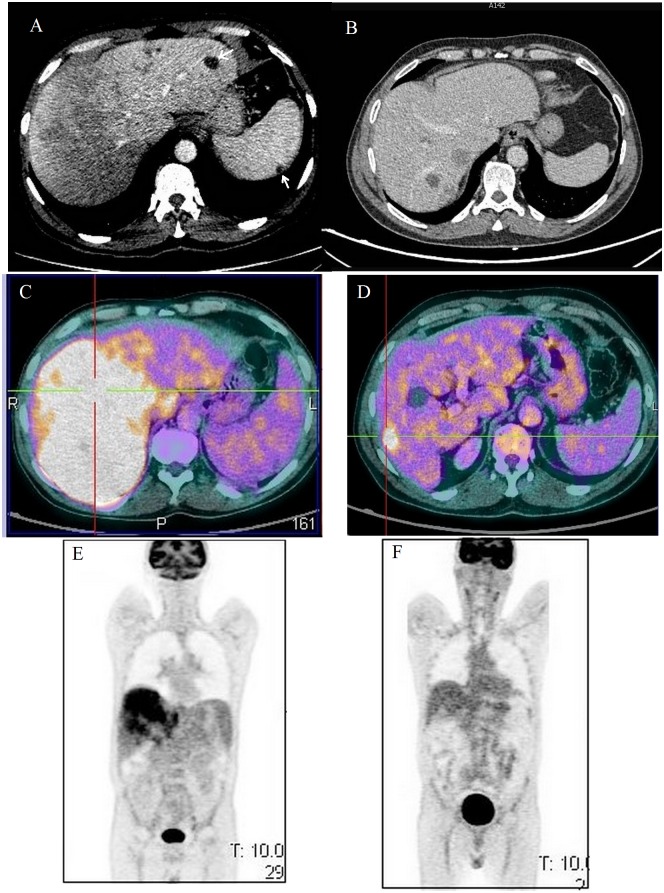
Imaging characteristics and evolution of hepatic toxocariasis. Abdominal computed tomography (CT) scan before **(A)** and 2 years after treatment **(B)**. Panel A shows diffuse patchy infiltrate of the liver with heterogeneous low-attenuated lesions and cystic lesions (white arrow); Cystic lesion of the spleen (white arrow). Positron emission tomography/computed tomography (PET/CT) before **(C)** and **(E)** and 2 years after treatment **(D)** and **(F)**.

A 61-year-old male was admitted in our hospital with a 1-month history of fatigue and loss of appetite associated with the following "B symptoms," namely, a moderate fever fluctuating up to 38.5°C, night sweats, and a 7-kg weight loss. The patient also complained of abdominal bloating and intermittent abdominal pain without any transit disorder, specifically diarrhea. He did not report coughing or vomiting, and he did not display any abnormal skin findings. His past medical history was unremarkable, including no allergies. He had no sick contacts and had not traveled recently out of Reunion Island. On physical examination, abdominal palpation revealed a slight tenderness in the right upper quadrant but no hepatomegaly. No enlarged lymph nodes were found. Laboratory investigations revealed several abnormalities including marked blood eosinophilia (10.1 G/L), hallmarks of blood inflammation, and cholestasis (Table 1). Abdominal ultrasonography (US) revealed multiple oval hepatic lesions with heterogeneous echogenicity. A computed tomography (CT) of the abdomen showed heterogeneous low-attenuation infiltrative lesions of the liver (Fig 1A).

**Table 1:** Characteristics of clinical and laboratory parameters.

	1 month before diagnosis	Hospitalization Day 1	Hospitalization Day 6	Hospitalizationafter 5-day treatment with albendazole Day 10	1 month after 10-day treatment with albendazole	6 months later	2 years later
weight (kg)	62	62	62	63	63.5	67	69
fatigue	+++	++++	++	+	+	-	-
fever (≥38.5°C)	*	*	-	-	-	-	-
CRP (mg/l) ^1^	108	56.6		135.7		10	0.8
eosinophilia (10^9^/l) ^2^	9.0	4.5	0.9	0.1	0.4	0.7	0.2
gammaglobulinemia (g/l) ^3^	26.7	32.6				11.69	
hemoglobin (g/dl) ^4^	12.3	7.3	11	9.6	12.5	16	14.9
LDH (UI/l) ^5^	551	810		401		300	254
platelets (10^9^/l) ^6^	341	34	91	89	191	226	229
total IgE (kIU/l) ^7^			1466				180
toxocara ELISA (OD) ^8^	1.23						1.28
ADAMTS13 activity (%) ^9^		<10				66	
anti-ADAMTS13 antibodies (UI/ml)^10^		190					
prednisone (mg/day) ^11^	0	0	60	60	40	10	0

**Abbreviations**: CRP, C-reactive protein; ELISA, enzyme-linked immunosorbent assay; Ig, immunoglobulin; LDH, lactate dehydrogenase; OD, optical density

++++ very intense, +++ intense, ++ moderate, + mild,—absent, * present,

^1^ normal range is 0–5 mg/l,

^2^ normal range is 0–0.4 G/l,

^3^ normal range is 8–13.5 g/l,

^4^ normal range is 12–16 g/dl,

^5^ normal range is 240–480 UI/L,

^6^ normal range is 150–400 10^9^/l,

^7^ normal range is 5–150 kIU/l,

^8^ normal value is <0.5 OD,

^9^ normal range is 50–150%,

^10^ normal value is <25 UI/ml,

^11^ treatment with steroid pulses from day 2 to month 6

Chest CT did not detect any abnormality. The patient underwent an extensive evaluation for underlying causes of hypereosinophilia, including screening for malignant, infectious, and autoimmune disorders (S1 Table). A high level of serum total immunoglobulin (Ig) E (Table 1) supported the presence of a tissue-dwelling helminthiasis; thus, immunodiagnostics of ascariasis, echinococcoses, fascioliasis, schistosomiasis, strongyloidiasis, toxocariasis, and trichinellosis were requested. Bone marrow section revealed eosinophil infiltration (26%) with normal immunohistochemical profile of B and T cells. 18F-fluorodeoxyglucose positron emission tomography/computed tomography (PET/CT) showed diffuse intense heterogeneous hypermetabolic activity in the liver standardized uptake value (SUV) max value = 10) but no primary tumor was seen (Fig 1C and 1E). Transthoracic echocardiography showed normal heart function without cardiomyopathy. While laboratory investigations were pending, the patient was discharged.

**Fig 2:**
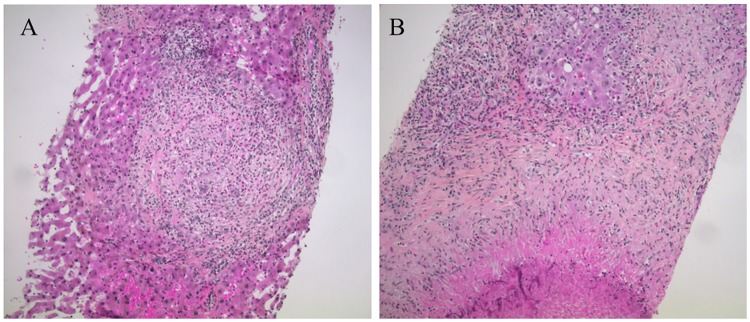
Histology of hepatic toxocariasis. **(A)** Hepatic granuloma with extensive infiltration of eosinophils. **(B)** Hepatocyte necrosis (hematoxylin and eosin staining, magnification x 100). Larvae were not observed. Ziehl, Periodic Acid Schiff, auramine, and Grocott’s stains were negative and immunochemistry ruled out lymphoma.

Three weeks later, he was readmitted in order to undergo a liver biopsy. Blood count results showed hemolytic anemia (7.3 g/dl), thrombopenia (34 G/l), and elevated schistocytes (0.5–0.7%). Coombs test (IgG and C3d) was positive and renal function was normal. Because Evans syndrome or thrombotic microangiopathy (TMA) was suspected, we started a corticosteroid therapy of 60 mg (total dosage) per day. Since strongyloidiasis is endemic in La Réunion, a single 12-mg round of ivermectin was given before corticosteroid treatment in order to prevent a drug-induced hyperinfection. On day 3 of corticosteroid therapy, the patient’s clinical condition improved dramatically and blood eosinophilia was 0.4 G/L. Findings on liver biopsy were highly suggestive of hepatic involvement due to VLM (Fig 2) [12]. Immunochemistry showed rare normal T and CD20-positive B cells without abnormality as part of the infiltration surrounding granulomatous lesions.

Eventually, the diagnosis of toxocariasis was confirmed by a positive enzyme-linked immunosorbent assay (ELISA), which detects antibodies against a second-stage larval excretory-secretory antigen, along with the finding of a typical 7-band pattern on Western blot (S1 Fig) [13]. The patient was treated with oral albendazole (400 mg twice daily for 10 days). Regarding his thrombocytopenia, ADAMTS13 activity was found to be less than 10% and the positivity of anti-ADAMTS13 autoantibodies confirmed the diagnosis of associated autoimmune TTP. The patient was discharged on day 13 due to an improved and stable condition.

## Follow-up and outcomes

Corticosteroid therapy was tapered then discontinued on month 6. A 2-year follow-up imaging with MRI and PET scan examination found an overall decline of the number of liver lesions along with a reduction of fluorodeoxyglucose uptake, although some lesions were still intensively active (SUVmax value = 6.7) (Fig 1B, 1D and 1F). Liver core biopsy carried out in month 29 showed similar findings to the initial biopsy, ruling out definitely any liver neoplasia.

## Discussion

As originally described by Beaver [3] then completed by further reports, VLM syndrome usually includes fever, loss of weight, cough due to irritation, wheezing, dyspnea, hepatomegaly, enlarged lymph nodes, lung infiltrates on chest radiography, and marked, persistent blood eosinophilia [1]. In our patient's case, the lack of any lung or lymphatic involvement therefore represented a puzzling difference, the origin of which could lie in an intense phenomenon of larval liver trapping [14]. Experiments carried out in animal models have demonstrated that the fate of *Toxocara* larvae entering the liver (see "Introduction") depends upon the size of the inoculum (number of ingested embryonated eggs) and the repetition of infection, in addition to genetic factors [15,16]. Following infection with smaller inoculums, larvae went freely through the liver. Conversely, in individuals repeatedly exposed to larger inoculums, challenge infections resulted in an intense larval trapping in the liver, where larvae were surrounded by eosinophil granulomas. Upon epidemiological questioning, our patient indicated he had frequent close contact with stray dogs and cats, a prominent risk factor [1], which suggested that the observed hepatic involvement and the lack of any major larval dissemination in his body could originate in the above-depicted pathophysiological process of liver trapping. However, it should be recalled that larvae retained inside granulomas remain alive for years, which underlines the need for anthelmintic therapy. Very little is known about the posttreatment evolution of hepatic granulomas, but they have been observed over 1 year posttreatment [17].

Autoimmune TTP is a life-threatening syndrome related to a severe deficiency of ADAMTS13 (<10%) secondary to anti-ADAMTS13 IgG antibodies [18]. Unlike systemic cancer, autoimmune disorders, and other systemic infections (viral, fungal, and bacterial), the occurrence of autoimmune TTP in the setting of a helminthic infection has not been reported to our knowledge. We cannot rule out a coincidental association between toxocariasis and autoimmune TTP, although it seems unlikely considering the low prevalence of autoimmune TTP.

According to the so-called hygiene hypothesis and also the coevolution theory, it has been hypothesized that helminth infections could protect against autoimmune diseases, particularly by shaping and tuning the immune system during the early life [19]. Thus, treatment of autoimmune diseases by helminth infections is being studied in clinical trials, even though there are immense conflicting data regarding the benefits versus harms of using live helminths [20,21]. However, some rare reports showed that, due to thus-far-unknown reasons, a helminth infection conversely can trigger or boost an autoimmune specificity [22]. This has been particularly observed with toxocariasis and trichinellosis [23]. Several mechanisms could explain the induction of autoimmunity by parasites, including molecular mimicry, polyclonal activation, and expansion of B cell clones generating autoantibodies. The B cell profile was normal in our case. This unique case of concurrent VLM and TTP highlights the complex relationship between helminth antigens and immunity and presents further evidence to question the “hygiene hypothesis.”

**Key learning points**

- Toxocariasis may induce autoimmune disease and must be considered as a possible cause of autoimmune TTP.
- Clinicians should consider the diagnosis of VLM despite the lack of any lung or lymphatic involvement.
- Hepatic toxocariasis was successfully treated with albendazole and corticosteroids.
- Clinical-radiological dissociation is observed in posttreatment evolution of hepatic granuloma.

## Supporting information

S1 Fig Positive Western blot of toxocariasis. Only low-molecular-weight bands from 24 kDa to 35 kDa are specific.
(TIF)

S1 Table Main paraclinical investigations performed for hypereosinophilia causes. (DOCX)
