# Supplementary figures and images for: Atypical presentation of hepatic visceral larva migrans mimicking cancer and associated with ADAMTS13 deficiency–mediated thrombotic microangiopathy: A first report from Reunion Island
Source: PLoS Negl Trop Dis. 2017 Jul 20;11(7):e0005617. doi: 10.1371/journal.pntd.0005617 (PMC5518999; doi:10.1371/journal.pntd.0005617)

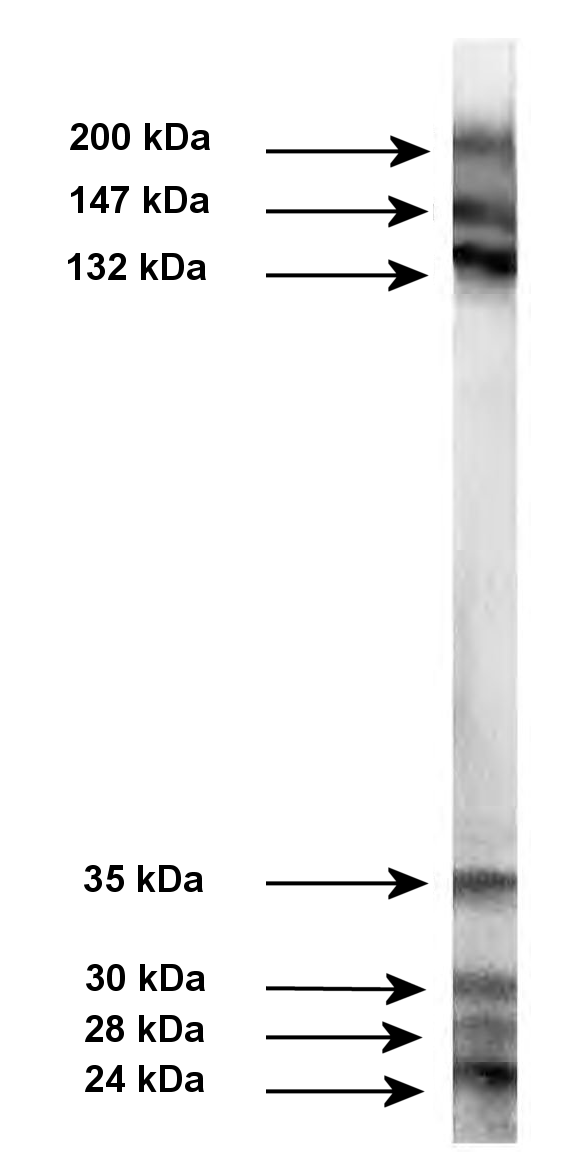

Supplement: S1 Fig — Only low-molecular-weight bands from 24 kDa to 35 kDa are specific. (TIF) [file pntd.0005617.s001.tif]
