# Supplementary material for: Atypical presentation of hepatic visceral larva migrans mimicking cancer and associated with ADAMTS13 deficiency–mediated thrombotic microangiopathy: A first report from Reunion Island
Source: PLoS Negl Trop Dis. 2017 Jul 20;11(7):e0005617. doi: 10.1371/journal.pntd.0005617 (PMC5518999; doi:10.1371/journal.pntd.0005617)
Supplement: S1 Table — (DOCX) [file pntd.0005617.s002.docx]

**S1 Table.** Main paraclinical investigations performed for hypereosinophilia causes.

| Investigations | Results |
| --- | --- |
| Malignancy | |
| Bone marrow biopsy | Eosinophilic infiltration of 26%. No evidence of lymphoproliferative or myeloproliferative neoplasms, no blast cells. |
| Flow cytometry  Cytogenetic analysis of bone marrow  FIP1L1-PDGFRA fusion gene screening  T cell clonality assessment | No clonal B-cell and no abnormal T-cell populations.  No translocation  No abnormalities  No clonal T-cell-receptor gene rearrangement |
| PET/CT scan (18-fluoro-2-deoxyglucose) | Intense heterogeneous hypermetabolic activity of the liver (SUVmax value= 10). Cystic non hypermetabolic hepatic lesions. Hypermetabolic lymphadenopathy on both sides of the diaphragm. No primary tumor found. |
| Tumor biomarkers: Alpha-fetoprotein, CA 19-9 | normal |
| Auto-immune disorders | |
| Antinuclear Ab, Anti-ENA, Anti-dsDNA Ab, ANCA Ab, antiphospholipid Ab, Anti-cardiolipin Ab, β2Gp1 Ab, anti-GBM Ab, C3 ,C4, CH50, Rheumatoid factor | normal |
| Infections | |
| Serology:  -Helminth:  *Toxocara*  *Strongyloides, Trichinella, Fasciola, Echinococcocus, Schistosoma, Ascaris suum.*  -Virus: HIV, HBV, HCV, CMV, EBV, HTLV-1  -Bacteria: Syphilis | Positive  Negative  Negative  negative |
| IGRAs | negative |
| Blood and urine cultures, parasitological examination of feces | negative |

**Note.**

Ab: antibodies; CMV: Cytomegalovirus; EBV: Epstein–Barr virus; HBV: Hepatitis B virus; HCV: hepatitis C virus; HTLV-1: Human t-cell lymphotropic virus type 1; IGRAs: Interferon-gamma release assays.
